# Response to Rodríguez-Cuadrado et al’s “Clinical, histopathologic, immunohistochemical, and electron microscopic findings in cutaneous monkeypox: A multicenter retrospective case series in Spain”

**DOI:** 10.1016/j.jdcr.2024.07.042

**Published:** 2024-09-13

**Authors:** Vina V. Ravichandran, Emily Coleman, Alexandria Brown, Erin Boh

**Affiliations:** Department of Dermatology, Tulane University School of Medicine, New Orleans, Louisiana

**Keywords:** herpes, histopathology, medical dermatology, mpox, Tzanck smear

*To the Editor:* Due to the 2022 outbreak, monkeypox (mpox) became a top differential when patients present with a flu-like prodrome followed by scattered papulovesicular or papulopustular lesions.^1^^,^^2^ There has been increased interest in specific clinical and histopathologic features to differentiate this disease from its mimickers, most notably herpes zoster virus or varicella zoster virus.^3^ We read the article by Rodríguez-Cuadrado et al that states that histopathologic findings were similar in all mpox cases studied.^1^ Mpox skin biopsies showed an epidermis with “necrosis and irregular hyperplasia at both sides” and “eosinophilic cytoplasmic inclusions (Guarnieri’s inclusion bodies) in keratinocytes of the upper layers of the epidermis with balloon cell change.”^1^ We describe the case of an HIV-negative patient in his 20s, presenting with papulopustular lesions on his face (Fig 1). The histopathology of one pustule showed viral inclusion bodies on hematoxylin and eosiin stain and positive serology, consistent with the diagnosis of mpox, while excluding other differentials, such as herpes simplex since lesional fluid herpes simplex virus1/2 polymerase chain reaction was negative. Interestingly, the Tzanck smear demonstrated multinucleated giant cells (Fig 2). Typically, multinucleated giant cells are not as frequently observed in Tzanck smears of mpox lesions in comparison to herpetic lesions. This case demonstrates that bedside diagnostic tools such as Tzanck smear are important yet may also confound the diagnosis given the presence of multinucleated giant cells in herpes zoster or herpes simplex lesions. Clinical pathologic correlation is key, and mpox lesions classically spread cephalocaudally and may also be present in the anal mucosa as was the case in our patient.

**Fig 1:**
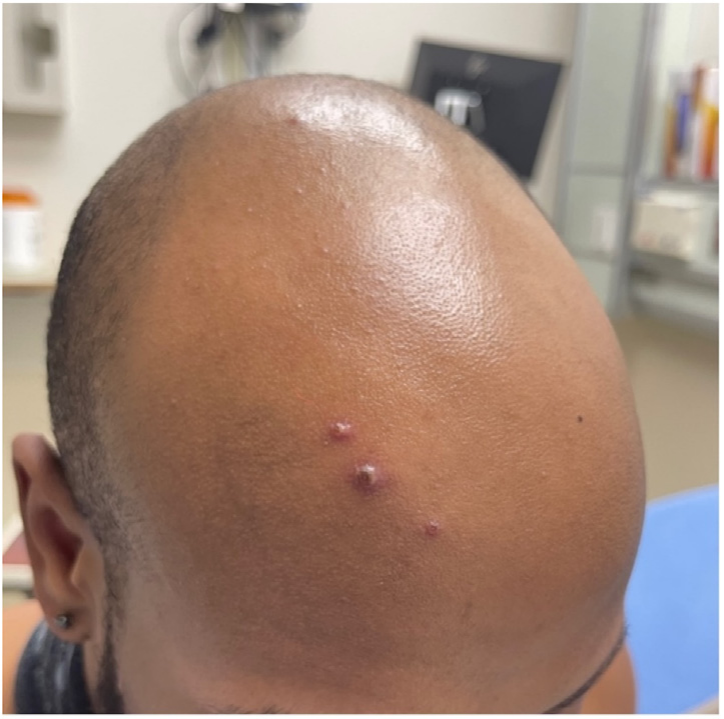
Mpox: Clinical presentation. *Mpox*, Monkeypox.

**Fig 2:**
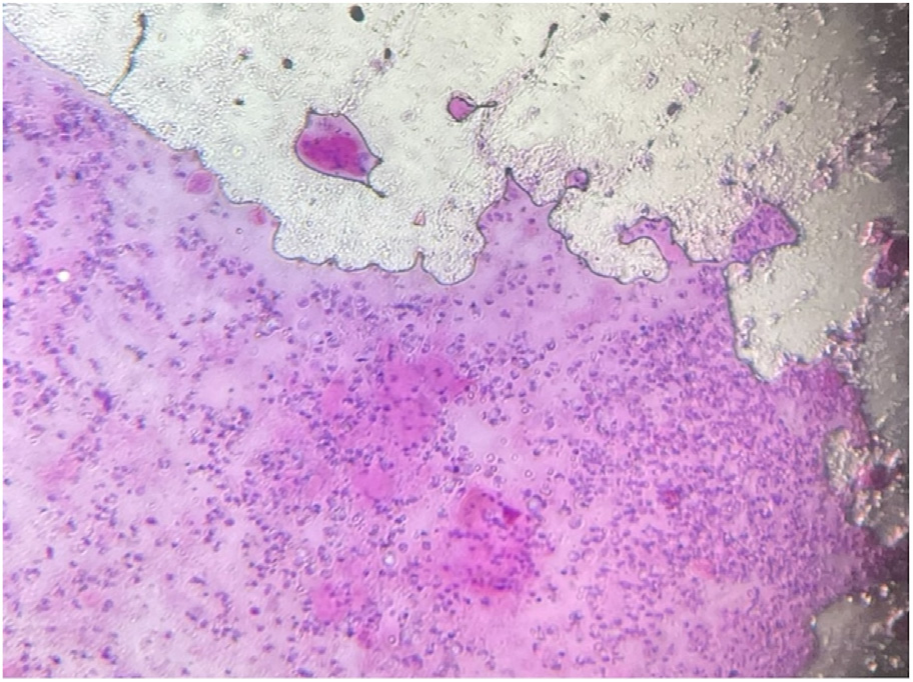
Mpox: Tzanck smear. *Mpox*, Monkeypox.

The majority of mpox cases have occurred in men who have sex with men, especially those who are HIV positive.^2^ About 40% of patients diagnosed with mpox also have HIV.^4^ Interestingly, our men who have sex with men patient was HIV negative. Although mpox is more prevalent in men who have sex with men and/or HIV positive patients, diagnosis should not be excluded in patients outside those populations, and HIV testing is paramount in any suspected mpox case.^4^

## Conflicts of interest

None disclosed.
